# Motion Compensated Structured Low-rank Reconstruction for 3D Multi-shot EPI

**DOI:** 10.1002/mrm.30019

**Published:** 2024-02-15

**Authors:** Xi Chen, Wenchuan Wu, Mark Chiew

**Affiliations:** 1 Department of Radiological Sciences, David Geffen School of Medicine at UCLA, Los Angeles, California, USA; 2 Wellcome Centre for Integrative Neuroimaging, FMRIB, Nuffield Department of Clinical Neurosciences, University of Oxford, Oxford, United Kingdom; 3 Physical Sciences, Sunnybrook Research Institute, Toronto, Canada; 4 Department of Medical Biophysics, University of Toronto, Toronto, Canada

**Keywords:** 3D multi-shot EPI, structured low-rank, motion compensation, phase variations

## Abstract

**Purpose:**

Three-dimensional (3D) multi-shot echo planar imaging (EPI) imaging offers several benefits including higher SNR and high isotropic resolution compared to two-dimensional single shot EPI. However, it suffers from shot-to-shot inconsistencies arising from physiologically induced phase variations and bulk motion. This work proposed a motion compensated structured low-rank (mcSLR) reconstruction method to address both issues for 3D multi-shot EPI.

**Methods:**

Structured low-rank reconstruction has been successfully used in previous work to deal with inter-shot phase variations for 3D multi-shot EPI imaging. It circumvents the estimation of phase variations by reconstructing an individual image for each phase state which are then sum-of-squares combined, exploiting their linear interdependency encoded in structured low-rank constraints. However, structured low-rank constraints become less effective in the presence of inter-shot motion, which corrupts image magnitude consistency and invalidates the linear relationship between shots. Thus, this work jointly models inter-shot phase variations and motion corruptions by incorporating rigid motion compensation for structured low-rank reconstruction, where motion estimates are obtained in a fully data-driven way without relying on external hardware or imaging navigators.

**Results:**

Simulation and in vivo experiments at 7T have demonstrated that the mcSLR method can effectively reduce image artefacts and improve the robustness of 3D multi-shot EPI, outperforming existing methods which only address inter-shot phase variations or motion, but not both.

**Conclusion:**

The proposed mcSLR reconstruction compensates for rigid motion, and thus improves the validity of structured low-rank constraints, resulting in improved robustness of 3D multi-shot EPI to both inter-shot motion and phase variations.

## 1 Introduction

Two-dimensional (2D) echo planar imaging (EPI), particularly 2D simultaneous multi-slice EPI, has been the workhorse of functional (fMRI) and diffusion MRI (dMRI) studies in recent years. However, three-dimensional (3D) EPI imaging is increasingly being explored as an alternative, especially in high resolution fMRI^1–9^ and dMRI^10–14^ at ultra-high fields. In addition to fMRI and dMRI, 3D EPI has also been used in quantitative parameter mapping^15–17^ and other structural imaging ^18,19^. Compared to 2D EPI, 3D EPI confers several advantages. First, 3D encoding offers improved SNR as the whole volume is excited repeatedly by every shot, enabling efficient signal averaging. Second, 3D EPI is less likely to run into specific absorption rate (SAR) constraints as the optimal flip angles for 3D EPI are much lower compared to 2D multi-slice imaging. Moreover, 3D EPI can achieve thin slices without being limited by RF profile imperfections, enabling high isotropic resolution. Finally, motion-induced spin history effects are less detrimental in 3D EPI compared to 2D EPI.

3D EPI typically uses a multi-shot acquisition strategy to sample the full 3D k-space for whole brain imaging. However, due to physiological fluctuations (e.g., respiration and cardiac pulsation) and subject movement, 3D multi-shot EPI imaging is vulnerable to shot-to-shot inconsistencies. These physiological fluctuations can cause image phase inconsistencies across shots which result in image artefacts. In dMRI, drastic inter-shot phase variations induced by subtle physiological motion (e.g., cerebrospinal fluid pulsation) during diffusion encoding can lead to severe ghost artefacts^20–22^, which presents a significant challenge for high resolution dMRI. In fMRI, phase variations are mainly contributed by physiologically induced *B*_0_ field fluctuations due to respiratory movements of the chest. Phase variations in multi-shot fMRI typically do not produce noticeable ghost artefacts in each image volume, but can lead to unwanted image magnitude fluctuations between volumes, which impairs the temporal stability of the time course^23^. As a result, the SNR benefit of 3D imaging is not fully realized in 3D multi-shot EPI fMRI, which might only be able to achieve a higher temporal signal-to-noise ratio (tSNR) than 2D single-shot EPI in low SNR, thermal noise dominated regimes ^4,24^. In addition, as the off-resonance effect scales with field strength, these inter-shot phase variations are more detrimental for fMRI at ultra-high fields such as 7T.

Previous methods for dealing with the shot-to-shot phase variations can be broadly divided into two categories depending on whether an explicit phase estimate is needed. Phase estimation-based methods typically incorporate phase maps into the forward model explicitly, which are estimated from additional MRI navigators or a data-driven procedure. Various navigator acquisition methods ^25–29^ have been developed to measure the global, 1D or 2D *B*_0_ field fluctuations in fMRI. Similar methods have also been developed to deal with the inter-shot phase variations in multi-shot dMRI ^13,30–37^. However, navigator acquisition techniques prolong acquisition time and reduce achievable temporal resolution, and can be particularly challenging when 3D navigators are acquired. Data-driven methods for phase variation estimation have also been demonstrated for multi-shot dMRI ^21,38–42^, which do not acquire additional navigators at the cost of extra scan time, but reconstructions can suffer from poor conditioning, particularly as the number of shots increases.

**Fig. 1:**
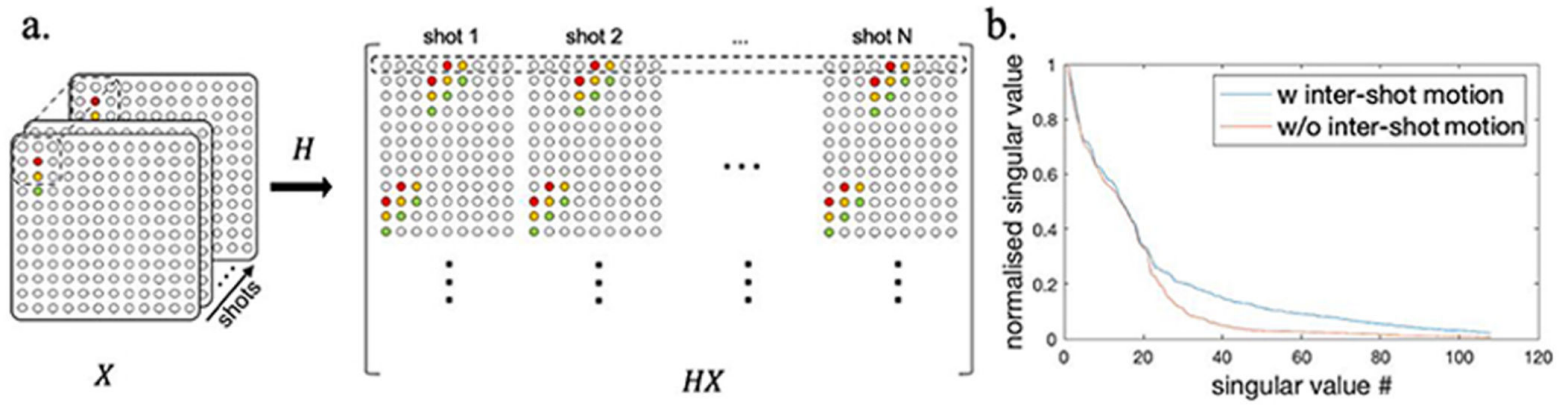
a) The construction of block-Hankel structured matrix from multi-shot k-space. The small patches of k-space from each shot selected by a sliding kernel are vectorized and concatenated to be a row vector of the block-Hankel structured matrix. X denotes the multi-shot k-space and the operator H constructs the block-Hankel structured matrix. b) The normalized singular values of two block-Hankel structured matrices constructed from simulated 3-shot 2D EPI datasets with and without inter-shot motion. The dataset with inter-shot motion was simulated with a 3° rotation between every two consecutive shots. Both datasets were also simulated with the same phase variation maps.

In contrast to methods relying on explicit phase estimates, a structured low-rank (SLR) based image reconstruction method MUSSELS ^22^ has been proposed to deal with the inter-shot phase variations for 2D multi-shot dMRI without the need of phase estimates, which has shown superior performance compared to explicit phase estimation based methods. MUSSELS addresses the shot-to-shot phase inconsistencies by reconstructing an individual image for each shot, which are sum-of-squared (SOS) combined to generate a final phase-insensitive reconstruction. In the MUSSELS approach, the k-space of all shots are jointly recovered by exploiting the linear dependencies between shots, which is formulated as a low-rank constraint on the block-Hankel structured matrix constructed from the multi-shot k-space (Fig.1a). In our previous work, we have adapted the structured low-rank reconstruction approach to deal with the phase inconsistencies for 3D multi-shot EPI in fMRI ^43^, which demonstrated that the proposed method is capable of improving tSNR in non-thermal noise dominated regimes.

In addition to physiologically induced phase variations, 3D multi-shot EPI acquisitions are also more vulnerable to bulk motion (i.e., head rotation and translation) than 2D single shot EPI due to the longer acquisition time for each image volume. Inter-shot motion in 3D multi-shot EPI can result in image artefacts and blurring arising from intra-volume, shot-to-shot inconsistencies, as well as misregistration between different volumes due to inter-volume inconsistencies. In addition, inter-shot motion is also problematic for the aforementioned SLR-based multi-shot EPI reconstruction, as they undermine the basic assumption that different shots share the same image magnitude and only differ in image phase. As such, the validity of the SLR constraint is compromised and the joint reconstruction of all the shots may not provide the expected benefit. Fig. 1b shows a simulation illustrating the impact of inter-shot motion on the singular value spectrum of the block-Hankel structured matrix representation of a multi-shot k-space, where multi-shot datasets with and without inter-shot motion are compared. The low-rank property of the block-Hankel structured matrix is compromised in the presence of inter-shot motion.

Previous intra-volume motion correction methods can be broadly divided into two categories depending on how motion parameters are estimated. One class of methods relies on additional MRI navigators or external hardware (e.g., optical tracking systems) to obtain motion estimates, which can be used for either prospective or retrospective motion correction ^44–49^. The other type of motion correction methods uses data-driven approaches to estimate motion parameters directly from the imaging data ^50–56^. The methods AMUSE ^52^ and SENDIMENT ^56^ estimate inter-shot macroscale motion and motion induced phase errors for 2D multi-shot dMRI from SENSE reconstructed shot images. Cordero-Grande et al. ^50^ proposed a method to jointly estimates the image and rigid motion parameters for multi-shot structural imaging by exploring the sensitivity encoding redundancy with an “aligned” SENSE-based reconstruction. This method is referred to as “mcSENSE” in this paper. The method TAMER ^54^ solves a similar joint image-motion optimization problem as mcSENSE with some efficiency optimizations. The method DISORDER ^51^ further improves the performance and efficiency of mcSENSE using distributed and incoherent sampling orders. More recently, DISORDER has been further developed to address pose-dependent *B*_0_ field changes for structural imaging at 7T ^57^. However, none of the existing motion correction methods compensate for physiologically induced phase variations for 3D multi-shot EPI.

In this work, we propose a motion compensated structured low-rank reconstruction framework for robust 3D multi-shot EPI imaging which jointly considers both inter-shot motion and phase variations. This method is termed mcSLR reconstruction hereinafter. It builds on the previous structured low-rank image reconstruction ^43^ which accounts for physiologically induced inter-shot phase variations, and further incorporates additional inter-shot motion compensation. Motion estimates are obtained in a fully data-driven way, and the joint estimation of motion and image are solved in an alternating fashion. Simulation and in vivo experiments at 7T have demonstrated that the proposed method can reduce motion artefacts and improve the robustness of 3D multi-shot EPI, which outperforms previous SLR based reconstruction which only considers inter-shot phase variations, and mcSENSE which only considers inter-shot rigid motion.

## 2 Methods

### 2.1 Formulation of the Optimization Problem

Since the number of shots can be quite large for 3D whole brain imaging, reconstruction of an individual 3D image from each shot can be extremely challenging even with SLR based reconstruction ^22^. Hence, the previous SLR reconstruction for 3D multi-shot EPI bins a small number of shots into a shot group and jointly reconstructs an image for each shot group. The low-rank constraint is enforced on the block-Hankel structured matrix constructed from the k-space of all shot groups to exploit their linear dependencies. Here, we propose to extend the SLR reconstruction framework to incorporate rigid-body motion transformations in the forward model to compensate for motion induced k-space inconsistencies. The reconstruction is formulated as the following optimization problem:

(Eq. 1) $$\underset{X,T}{\text{argmin}}||AFSTX-Y|{|}_{2}^{2}+\lambda ||HFX|{|}_{*}$$

Where *X*: [*x*_1,_ *x*_2_, … *x*_*n*_] consists of 3D images for *n* shot groups. *T* accounts for the rigid motion transformation with six motion parameters (i.e., 3 translation parameters and 3 rotation parameters). Rigid motion transformation was performed using convolution-based interpolation ^50,58^, which allows for rotation without regridding and thus better preserves the image resolution. Details of motion transformation can be found in the Appendix A. The operator *S* applies coil sensitivity encoding, *F* performs Fourier transform, *A* denotes the k space sampling operator, and *Y* is the acquire k-space data. The operator *H* constructs the block-Hankel structured matrix from the k-space data of all shot groups *FX*, and *λ* is the regularization parameter balancing the data consistency and SLR constraints.

### 2.2 Solution to the Optimization Problem

**Fig. 2:**
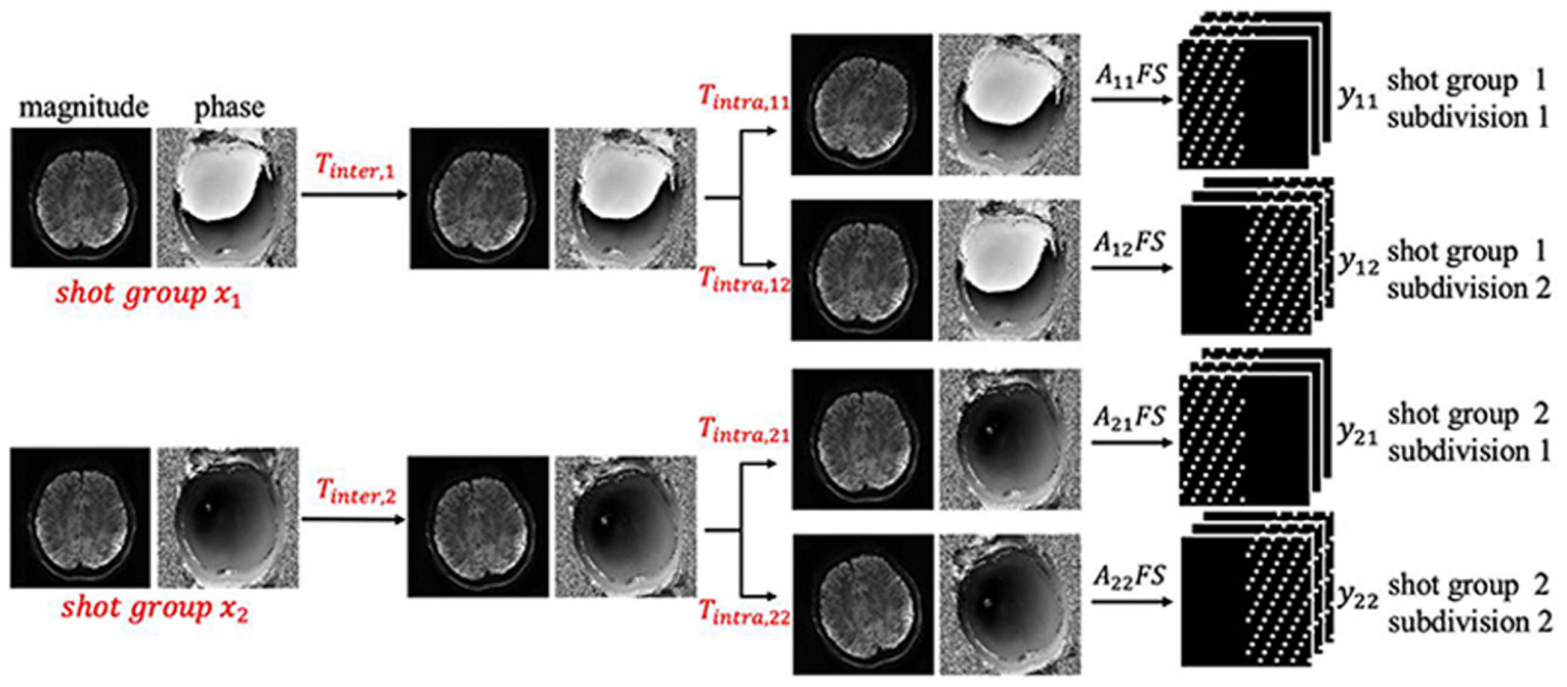
The forward model of the mcSLR reconstruction. The images of different shot groups (e.g., *x*_1_, *x*_2_) to be reconstructed are assumed to be aligned. The inter-shot group motion operator *T_inter,i_* transforms the aligned image of each shot group *x_i_* to its relative position. The intra-shot group motion operator *T_intra,ij_* then maps *T_inter,i_x_i_* to the motion state corresponding to the *j_th_* temporal subdivision of that shot group. *A_ij_* denotes the sampling mask of the *j_th_* temporal subdivision in the *i_th_* shot group, and *y_ij_* corresponds to its k-space data.

The proposed method jointly estimates the multi-shot group images, *X*, and motion parameters, *T*, in a fully data driven way, without relying on an external hardware or MRI navigators to acquire motion traces. As shown in Fig. 2, the intra-volume motion *T* is further decomposed into *inter*-shot group motion *T*_*inter*_ and *intra*-shot group motion *T*_*intra*_ using a hierarchical strategy which can be estimated efficiently by different methods. The inter-shot group motion *T*_*inter*_ accounts for the motion between the images of different shot groups, and the intra-shot group *T*_*intra*_ accounts for the motion between different temporal subdivisions consisting of one or more shots within each shot group. The optimization is solved in an alternating fashion where the three unknowns *X, T*_*intra*_, *T*_*inter*_ are updated by solving three subproblems sequentially within each iteration.

#### Subproblem 1-Estimate X by solving a pseudo 3D SLR optimization

The images of all shot groups *X* are estimated in subproblem 1 by solving the SLR optimization with the motion estimates from the last iteration. The block-Hankel structured matrix constructed from the 3D dataset using a 3D kernel has an extremely large size (> 10^6^ patches with patch size ~6x6x6), making it computationally challenging to work with. Therefore, we propose a pseudo 3D structured low-rank constrained reconstruction. In this formulation, the low-rank constraint is applied on the block-Hankel structured matrix constructed from the k-space of each 2D image slice along the *x, y* or *z* direction. The optimization with this pseudo 3D structured low-rank constraint is reformulated as:

(Eq. 2) $$\underset{X}{\text{argmin}}||AFS{\hat{T}}_{intra}{\hat{T}}_{inter}X-Y|{|}_{2}^{2}+\lambda \sum_{l=1}^{N_{l}}||H_{2D}FX_{l}|{|}_{*}$$

Where ${\hat{T}}_{intra}$ and ${\hat{T}}_{inter}$ are the motion estimates obtained from the last iteration. *X*_*l*_ denotes a 2D image slice extracted from *X* along the chosen direction, and *l* is the slice index. *N*_*l*_ is the number of slices along the chosen direction. Note that *FX*_*l*_ corresponds to the k-space of a 2D image slice, not a 2D plane sampled from a 3D k-space. *H*_2*D*_ builds the block-Hankel structured matrix from a 2D k-space using a 2D kernel, which has a much smaller size (~10^4^ patches with a patch size ~6x6). This optimization subproblem is solved by ADMM as in the previous SLR reconstruction method ^43^.

#### Subproblem 2 - Estimate T_inter_ using image registration

Since the images associated with different shot groups are available after solving subproblem 1, the inter-shot group motion parameters can be estimated using image-based registration methods. In this work, the image registration method FLIRT provided in the FSL toolbox ^59–61^ was used with Correlation Ratio as the cost function. The image from the first shot group is chosen as the reference volume. In each iteration, the images from all other shot groups are registered to the reference to calculate the residual motion Δ*T*_*inter*_ between them, which is used to update the inter-shot group motion estimates.

#### Subproblem 3 - Estimate T_intra_ by solving the data consistency constraint

The intra-shot group motion parameters are estimated by solving the data consistency constraint which is formulated as:

(Eq. 3) $$\begin{array}{c} \underset{T_{intra,ij}}{\text{argmin}}||{\text{A}}_{ij}FST_{intra,ij}{\hat{T}}_{inter,i}{\hat{x}}_{i}-y_{ij}|{|}_{2}^{2}\\ \forall j=1,\ldots m;\;i=1,\ldots n \end{array}$$

Where *i* is the shot group index and *j* is the temporal subdivision index within each shot group. ${\hat{x}}_{i}$ and ${\hat{T}}_{inter,i}$ are the estimated image and inter-shot group motion corresponding to the *i*_*th*_shot group by solving subproblems 1 and 2. *A*_*ij*_ and *y*_*ij*_ are the sampling mask and the acquired k-space data of the *j*_*th*_ subdivision in the *i*_*th*_ shot group, respectively. Eq. 3 is solved by a modified Levenberg–Marquardt (LM) algorithm ^62,63^ based on the MATLAB implementation provided in mcSENSE ^50^. Implementation details can be found in Appendix B.

To speed up convergence of the optimization, *T*_*intra*_ is initialized using mcSENSE by reconstructing a single combined shot group consisting of all shots regardless of the inter-shot phase variations. This motion initialization is also used in the mcSENSE reconstruction of each shot group (without further updating motion estimates) to obtain the initialization of *X*.

### 2.3 Experiments

Simulation and in vivo experiments at 7T were performed to validate the performance of the proposed mcSLR reconstruction, which was evaluated by comparing to SENSE, mcSENSE and the previous SLR reconstruction. All subjects were scanned with informed consent under a technical development protocol approved by the local ethics committee. All in vivo data were collected on a Siemens Magnetom 7T scanner (Siemens Healthineers, Erlangen, Germany) with a 32-channel head-only receive coil (Nova Medical, Wilmington, MA, USA). A 3D gradient recalled echo EPI sequence with “seg-CAIPI” sampling trajectory which was proposed in conjunction with the previous SLR reconstruction^43^ was used in both simulation and in vivo experiments. The seg-CAIPI trajectory uses Δ*k*_*z*_-blipped CAIPI sampling pattern with an interleaved ordering along the *k*_*z*_/shot dimension, which provides a roughly uniform under-sampling pattern for each shot group. The sampling mask used in this work is shown in Supporting Fig. S1. The acceleration factor along the *k*_*y*_ and *k*_*z*_ dimension is *R*_*y*_ × *R*_*z*_ = 2 × The 3D whole brain acquisition consists of 96 slices along the *k*_*z*_ direction and the total number of shots is 48. The number of shot groups used for SLR and mcSLR is 4 (i.e., 12 consecutive shots are binned together to form a single shot group). Coil compression ^64^ was performed on the in vivo data for efficient computation. Coil sensitivity maps were calculated by a reference scan using the ESPIRiT implementation provided in the BART toolbox^65^. The Nyquist ghosting was corrected using the 3-line reference scan to perform a conventional linear phase correction. The kernel size of the block-Hankel transformation was chosen empirically to be 6 × 6. The 2D block-Hankel structured matrices were constructed from each 2D *k*_*y*_ − *k*_*z*_ k-space plane. *λ* = 0.1 was used for the simulations, and *λ* = 0.06 was used for the in vivo data for both SLR and mcSLR reconstructions. For mcSLR, the number of ADMM iterations in subproblem 1 was set to 5 for simulation and 1 for in vivo experiments, the number of LM iterations in subproblem 3 was set to 5. The outer loop of mcSLR that alternates between the three subproblems was stopped when it reached the maximum number of iterations (100 for the simulation data and 30 for the in vivo data) or when the relative change of the output image was smaller than 10^−3^. The SLR reconstruction used a matched stopping criterion. The mcSENSE reconstruction was stopped when it reached a maximum of 100 iterations or when the relative change of the output image was smaller than 10^−3^. The reconstruction was implemented in MATLAB R2020a (MathWorks, Inc.), and source code is available at https://github.com/XChen-p/mcSLR.

### Simulation Experiments

A 2D *k*_*y*_-*k*_*z*_ slice of a 3D multi-shot EPI dataset with inter-shot phase variations and in plane motion was simulated. The 2D slice was extracted after an inverse Fourier transform of the 3D dataset along the *k*_*x*_ direction. The 2D phase variation maps and ground truth image were obtained from a single shot EPI time series acquired at 7T with 1.5mm isotropic resolution. The matrix size of the 2D slice was 140 × 96 and TE/TR=20/40ms. Continuous motion traces for 48 shots were generated by linear interpolation from 8 discrete motion samples, which were randomly chosen from a uniform distribution within [−1.5, +1.5] degrees for in-plane rotation and [−1.5, +1.5] mm for 1D translation along the *k*_*y*_ direction. A time course consisting of 16 volumes was simulated and the tSNR of the image series reconstructed by different methods were compared. Note all time courses underwent image registration by MCFLIRT provided in FSL toolbox ^60^ prior to tSNR calculation to remove the contribution of residual inter-volume misregistration to the observed temporal variance. Each temporal subdivision consists of 1 shot and the temporal resolution of motion estimates is 40ms.

### In vivo Experiments

In vivo experiments were performed on three healthy subjects to validate the performance of the proposed mcSLR reconstruction. One subject was instructed to remain still, and the other two subjects were instructed to perform head rotations during the scan. Three levels of motion were performed, including mild motion with head rotation smaller than 2.5°, medium motion with head rotation between 2.5° and 5° and large motion with head rotation larger than 5°. The imaging parameters were: 1.8mm isotropic resolution, matrix size = 116 × 116 × 96, TE/TR = 23/55ms. Each temporal subdivision consisted of 6 shots and the temporal resolution of motion estimates was 330 ms (i.e., one motion estimate for every 6×TR).

## 3 Results

### Simulation Experiments

**Fig. 3:**
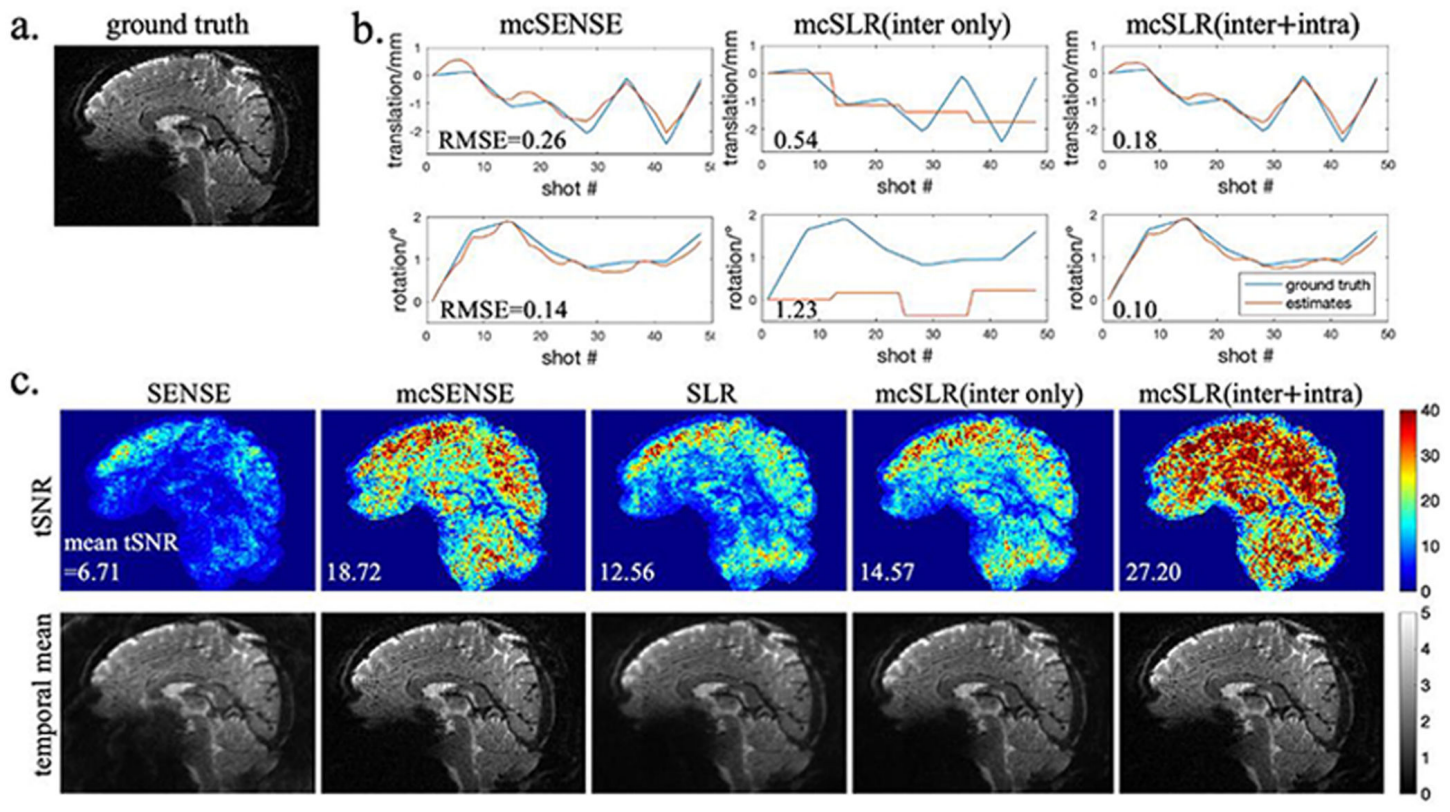
The comparison of different reconstruction methods in the simulation experiment. a) The ground truth magnitude image. b) The motion estimates of mcSENSE and mcSLR from an example volume. The RMSE of the motion estimates is shown in each subfigure respectively. c) The tSNR maps and temporal mean magnitude images of different reconstruction methods. The mean tSNR value is shown in each tSNR map respectively. The time course was registered with MCFLIRT prior to tSNR calculation.

Fig.3 shows the reconstruction results of the simulation experiment, where we compare different reconstruction methods including the mcSLR reconstruction with and without intra-shot group motion estimation. Fig.3a shows the ground truth magnitude image. Fig.3b shows the motion estimates from an example volume, and the root mean square error (RMSE) of the motion estimates is shown in each subfigure respectively. It is shown that mcSLR with both inter and intra shot group motion estimation has a higher motion estimation accuracy than mcSENSE and mcSLR with only inter-shot group motion estimation. The mean RMSE of the translation and rotation parameters estimated by mcSLR over 16 volumes are 0.15mm and 0.14°. Fig.3c shows the temporal mean images and tSNR maps of the different reconstruction methods, and the mean tSNR across the brain is reported in each subfigure respectively. Due to uncorrected subject motion, both SENSE and SLR have a blurry temporal mean image and a low tSNR. In comparison, mcSENSE improves the effective resolution of the temporal mean image and tSNR by incorporating motion compensation. Similarly, mcSLR with only inter-shot group motion estimation partially restores the spatial resolution and achieves a slightly higher tSNR than SLR, whereas mcSLR with both inter- and intra-shot group motion estimation largely gains the image resolution back and achieves a 117% higher mean tSNR than SLR. Despite comparable sharpness of the temporal mean images, mcSLR also achieves a 45% higher tSNR than mcSENSE, which can be partly attributed to its additional compensation of inter-shot group phase variations.

### In vivo Experiments

**Fig. 4:**
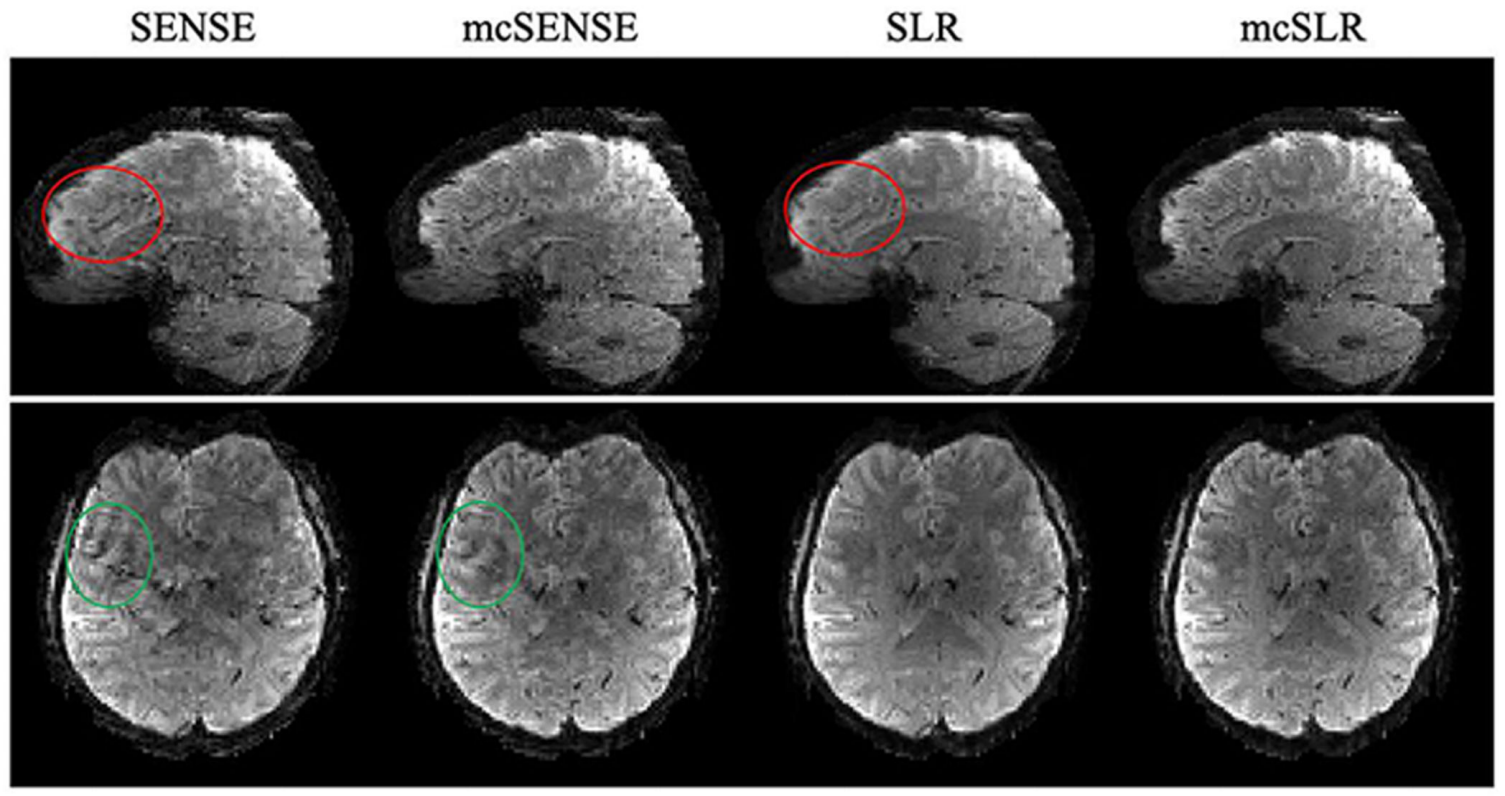
The comparison of different reconstruction methods on one volume with mild motion. The red ellipses in the sagittal view indicate the blurring of SENSE and SLR reconstructions. The green ellipses in the axial view indicate the residual artefacts of SENSE and mcSENSE reconstructions.

The reconstruction results of SLR and mcSLR on the motion-free dataset are shown in Supporting Fig. S2. The images reconstructed by these two methods do not show any noticeable differences, which suggests that mcSLR does not introduce any bias in the motion-free scenario. Fig. 4 shows the results of different reconstruction methods on one volume with mild motion. The red ellipses in the sagittal view indicate the blurring artefacts in SENSE and SLR reconstructions, which are not apparent in mcSENSE and mcSLR reconstructions. The green ellipses in the axial view indicate the residual artefacts of SENSE and mcSENSE reconstructions, likely due to signal cancellation from inter-shot phase variations. The proposed mcSLR method demonstrates overall the best performance. The motion estimates of mcSENSE and mcSLR for each subdivision are shown in Supporting Fig. S3, and they are in good agreement in the primary motion dimensions: rotation in the *y* − *z* plane and translation along the *y* direction. Note the motion estimates of mcSLR are shown as the combination of *T*_*inter*_ and *T*_*intra*_.

**Fig. 5:**
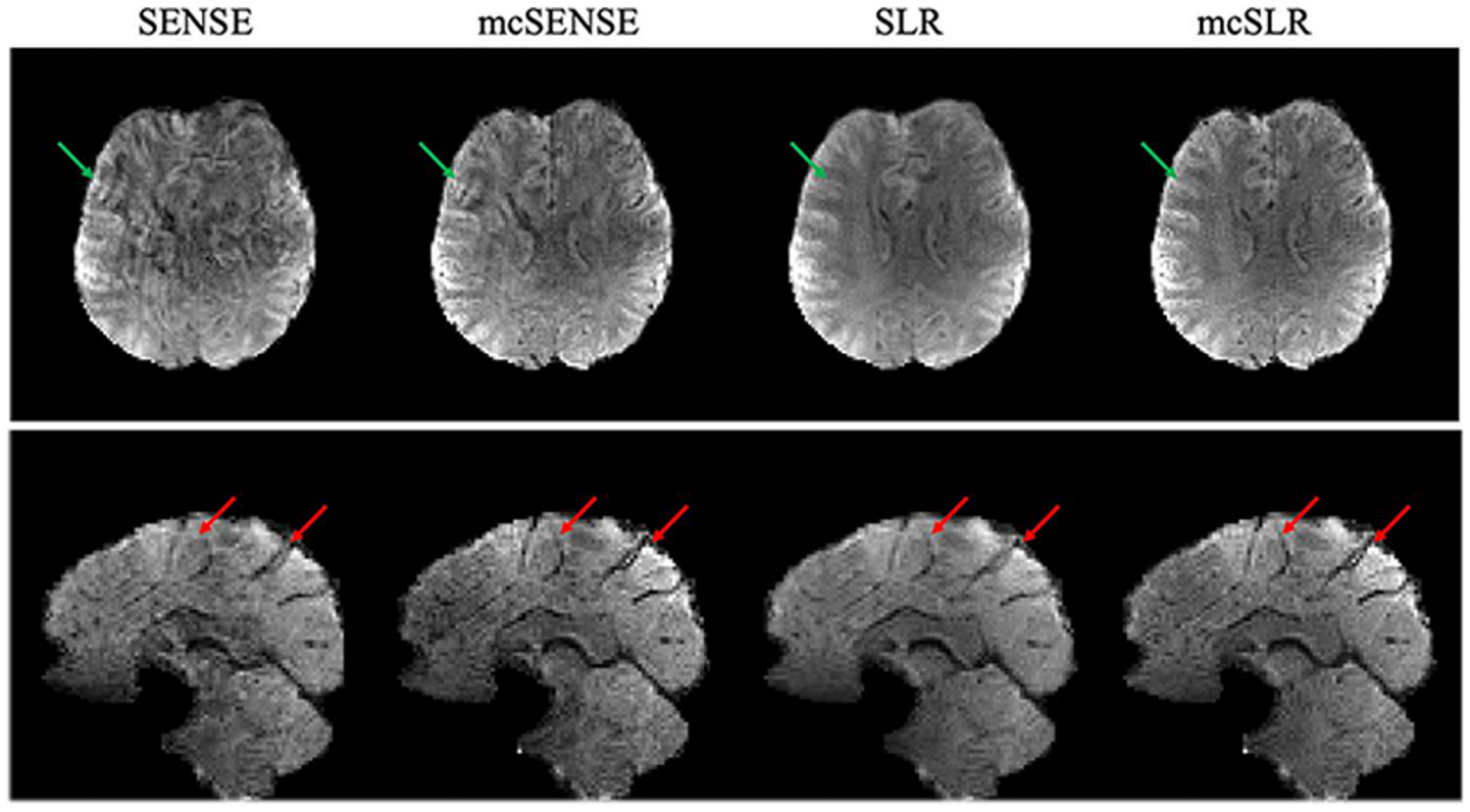
The comparison of different reconstruction methods on one volume with mild motion. Both axial view and sagittal view are shown. The green arrows in the axial view indicate artefacts in SENSE and mcSENSE reconstructions. The red arrows in the sagittal view indicate blurring in SENSE and SLR reconstructions.

Fig. 5 shows the results of one volume with mild motion. In this case, mcSENSE reduces the aliasing and blurring compared to SENSE reconstruction, but it still retains some artefacts as indicated by the green arrows, likely due to uncorrected inter-shot phase variations. The result of SLR reconstruction contains no severe aliasing artefacts as in SENSE or mcSENSE, but suffers from motion induced blurring as indicated by the red arrows. In addition, the phase variations between different shot groups might also contribute to the severe artefacts of SENSE and mcSENSE. The proposed mcSLR method further improves the sharpness compared to SLR and shows superior image quality compared to mcSENSE. The motion estimates of mcSENSE and mcSLR for each subdivision are shown in Supporting Fig. S4, and they are in good agreement.

**Fig. 6:**
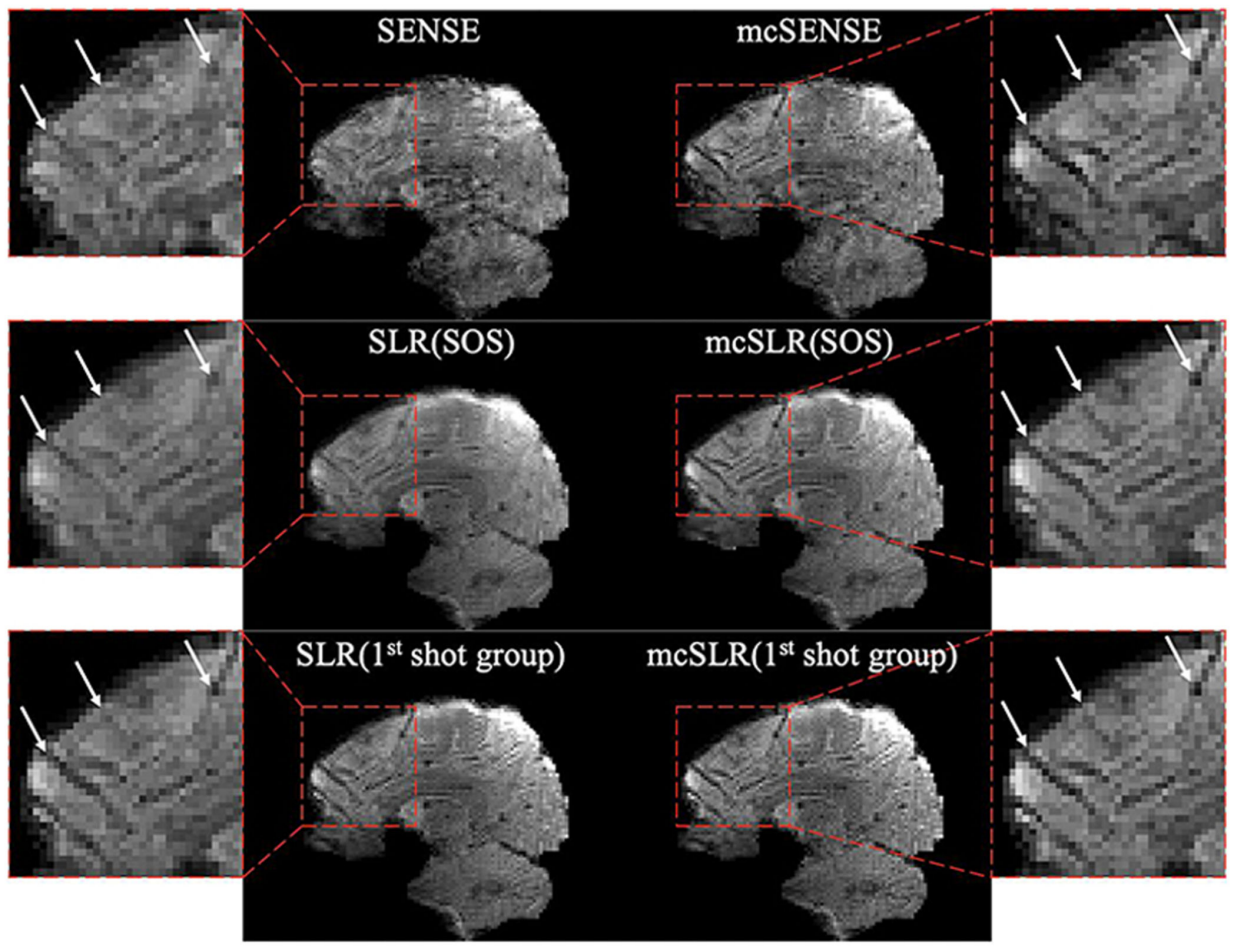
The comparison of different reconstruction methods on one volume with medium motion. The white arrow highlights small structures with varying sharpness by different reconstruction methods. For SLR and mcSLR, both the SOS combined image of all shot groups, and the image of the 1^st^ shot group are shown.

Fig. 6 shows the results of one volume with medium motion reconstructed by different methods. For SLR and mcSLR, both the SOS combined image of all shot groups, and the image of the first shot group are shown. By accounting for inter-shot motion, mcSENSE offers improved sharpness compared to the SOS combined image of SLR reconstruction, but it suffers from more image artefacts, particularly in the cerebellum, which is likely due to inter-shot phase variations. The first shot group image of mcSLR provides better delineation of fine structures than the first shot group image of SLR, which indicates successful intra-shot group motion correction. The SOS combined image of SLR is blurrier than its first shot group image, indicating uncorrected inter-shot group motion. In contrast, the first shot group image and the SOS combined image of mcSLR reconstruction demonstrate good alignment and comparable sharpness, which suggests successful inter-shot group motion correction. The results show that both intra- and inter-shot group motion correction contribute to the improved image quality of mcSLR over SLR.

**Fig. 7:**
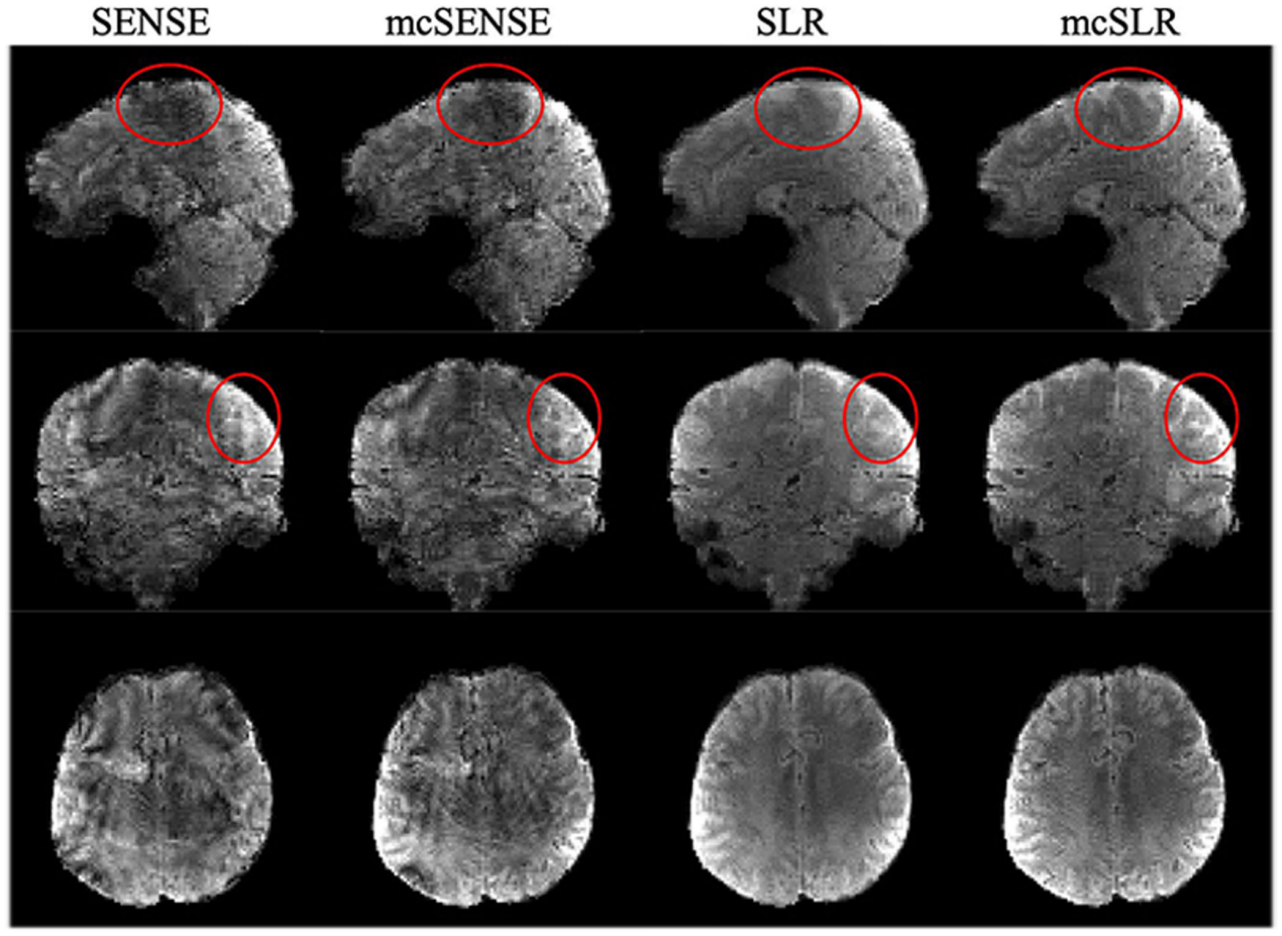
The comparison of different reconstruction methods on one volume with medium motion. The red ellipses highlight the improvement achieved by the proposed mcSLR reconstruction.

Fig. 7 shows the results of another example volume with medium motion. In this case, mcSENSE failed to achieve an improvement compared to SENSE. The results of SLR reconstruction have better image quality but strong blurring and poor contrast between grey matter and white matter. In contrast, the results of mcSLR reconstruction still show improved sharpness and better contrast that are necessary to disentangle small structures, highlighted by the red ellipses.

**Fig. 8:**
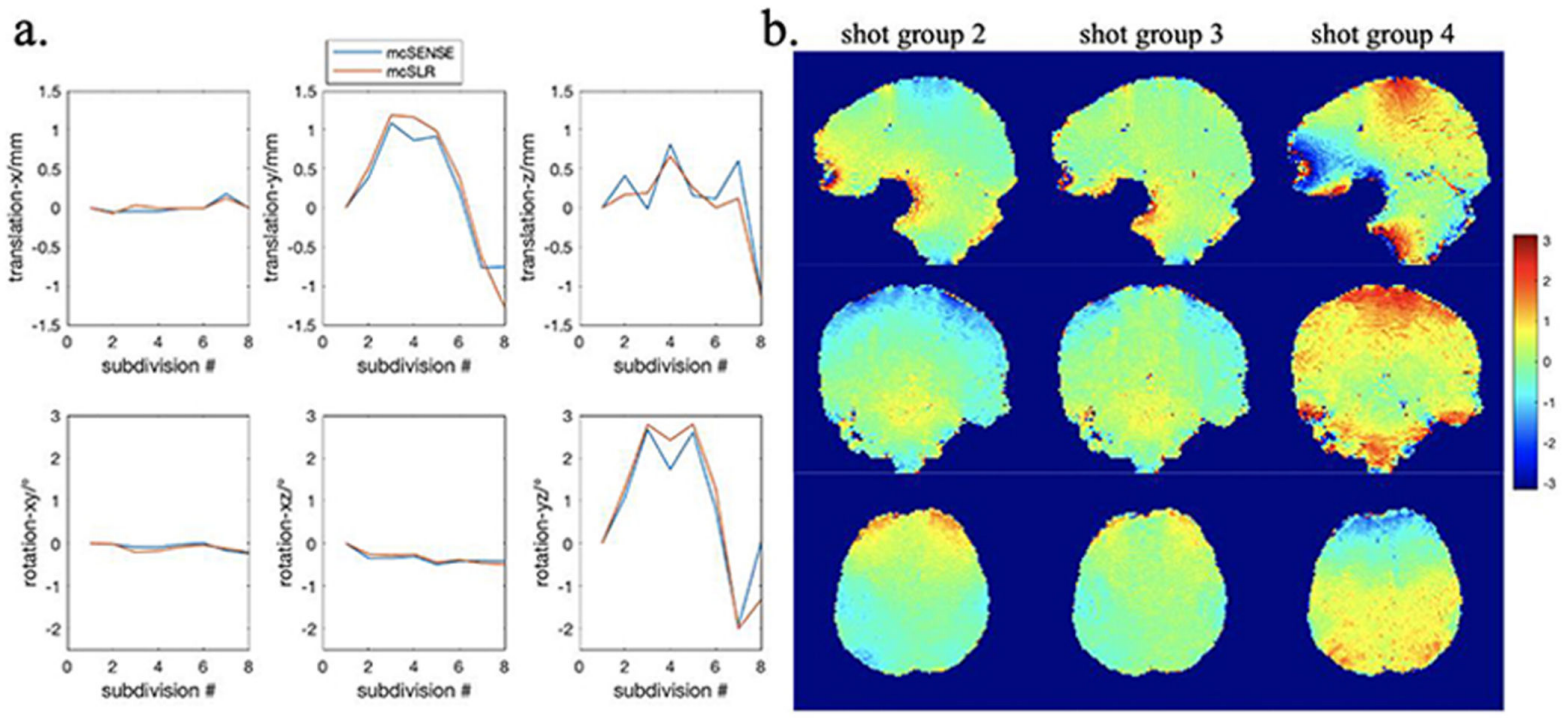
a) The motion estimates of mcSENSE reconstruction and mcSLR of the results shown in Fig. 7. b) The phase differences of different shot groups relative to the first shot group by mcSLR reconstruction of the results shown in Fig. 7.

The motion estimates of mcSENSE and mcSLR of the results shown in Fig. 7 are compared in Fig. 8a. They are in good agreement in the primary motion dimensions: rotation in the *y* − *z* plane and translation along the *y* and *z* directions. The phase differences of three shot groups relative to the first shot group by mcSLR are shown in Fig. 8b, where the phase variations between different shot groups can be observed. A tSNR comparison calculated across 15 volumes is shown in Supporting Figure S5. The mean tSNR of mcSLR is much higher than mcSENSE but slightly lower than SLR. However, the temporal mean image of SLR is blurrier than mcSLR (e.g., regions indicated by the red ellipse), which might lead to an inflated tSNR due to implicit spatial filtering effects.

**Fig. 9:**
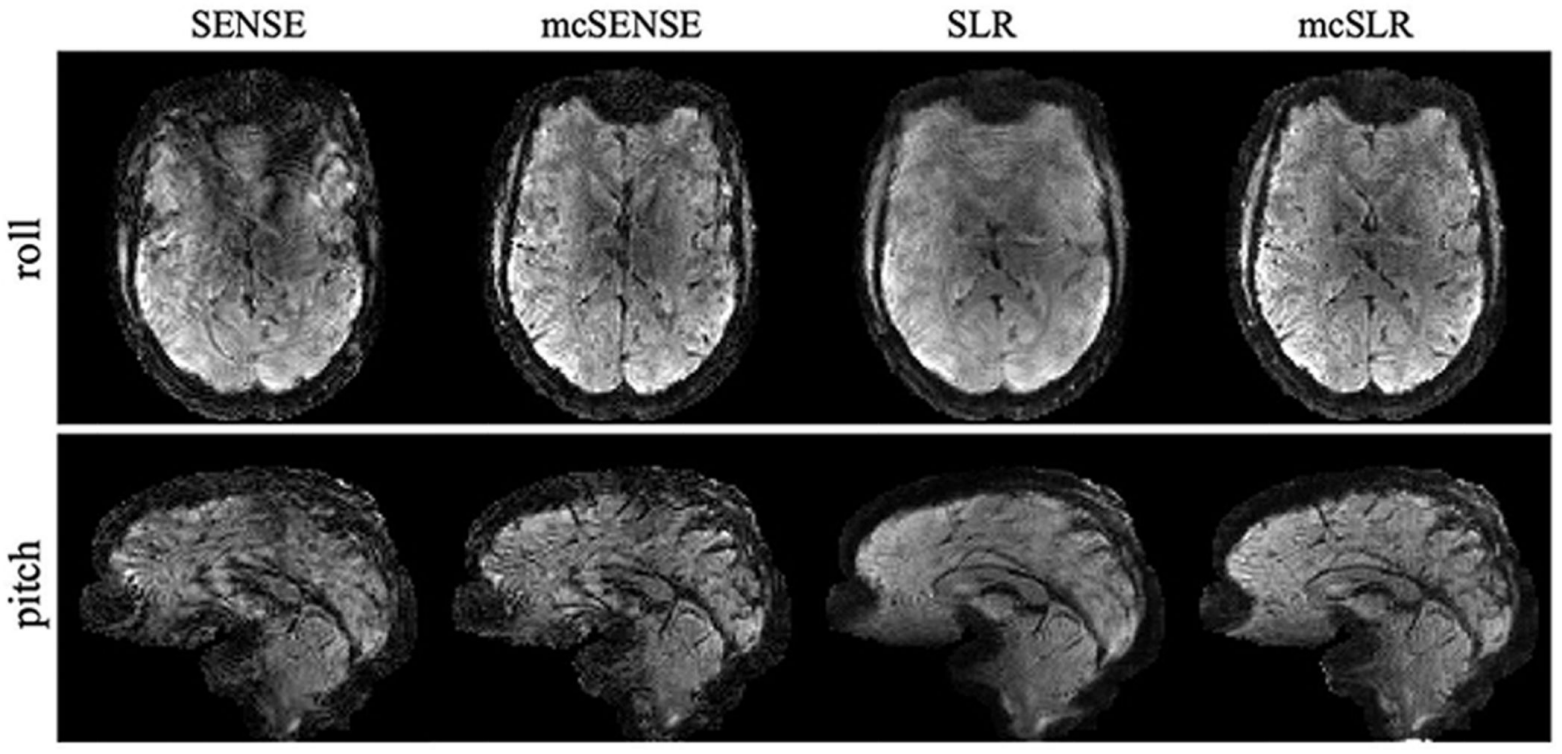
The comparison of different reconstruction methods on two volumes with roll and pitch rotations respectively at a comparable medium motion level.

**Fig. 10:**
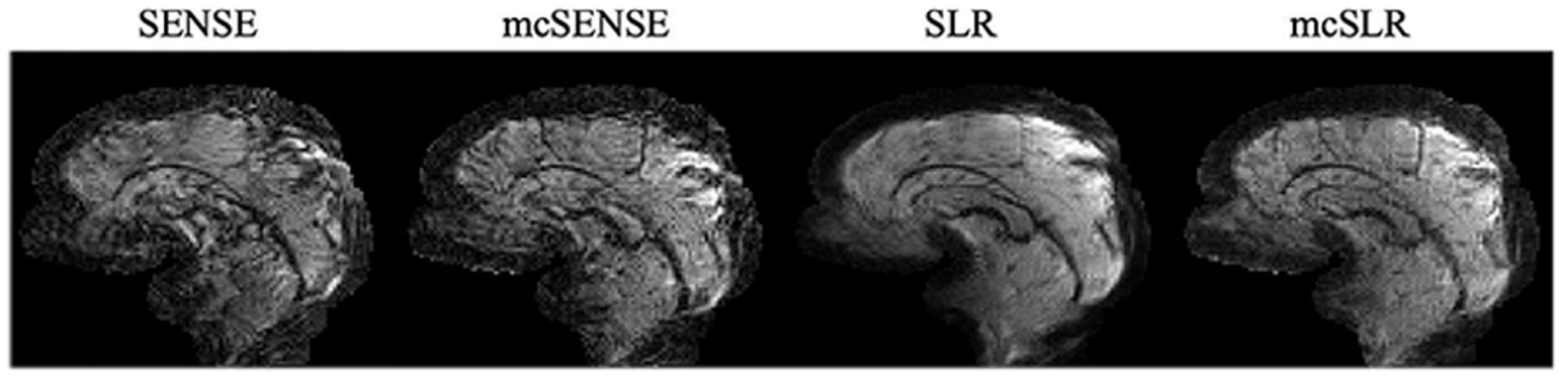
The comparison of different reconstruction methods on one volume with large motion.

Fig. 9 shows the results obtained on another subject where two types of motion, pitch (rotation around the left–right axis) and roll (rotation around the posterior-anterior axis) at a similar medium motion level are compared. The mcSENSE reconstruction performs worse with pitch rotation compared to roll rotation, as pitch motion likely induces stronger phase variations. However, the proposed mcSLR reconstruction achieves consistently good performance in both two cases. Fig. 10 shows the results of the same subject with large motion. The mcSLR reconstruction is still able to achieve an improvement compared to other methods when the rotation is up to around 8°. The motion estimates for Figs 9 and 10 are shown in Supporting Figs. S6 and S7, respectively.

## 4 Discussion

In this work, a motion compensated structured low-rank reconstruction framework for 3D multi-shot EPI is proposed, which improves its robustness to both inter-shot motion and phase variations. Simulation and in vivo experiments have shown that the proposed method can reduce image artefacts and improve the temporal stability of 3D multi-shot EPI in the presence of rigid head motion and physiologically induced phase variations. The proposed method can be particularly useful for 3D EPI fMRI, where the temporal stability across the long acquisition window is crucial for high sensitivity of subtle signal activations.

The proposed solution to inter-shot inconsistencies is built on the SLR framework which addresses inter-shot phase variations ^22,43^. The basic idea of SLR reconstruction for 3D multi-shot EPI is to reconstruct an individual image for each shot group, which has fewer signal variations due to a reduced time window of signal integration compared to the entire volume. To deal with the higher under-sampling factor of each shot group (compared to the combination of all shots), the SLR method jointly reconstructs all shot groups to exploit their linear dependencies. This low-rank property of the block-Hankel structured matrix is based on the assumption that different shot groups only differ in image phase while having consistent image magnitude. However, this assumption is clearly no longer true in the presence of inter-shot motion. Thus, SLR reconstruction with motion compensation can not only reduce motion artefacts, but also improve the validity of the structured low-rank constraint for the correction of phase variations.

The proposed mcSLR framework considers motion and phase variations at different time scales. The motion is considered at the intra-shot group level (i.e., between subdivisions within a shot group), as motion can be more random and less coherent compared to the physiologically induced phase variations. Also, the characterization of rigid motion for each motion state only needs six parameters instead of a full image needed to capture each shot-group’s unique phase distribution, making it feasible to estimate rigid motion from a small amount of data. As the physiologically induced phase variations are expected to be temporally coherent due to their dependence on the respiratory cycle, they are resolved only at the inter-shot group level. This improves the conditioning of the reconstruction, as the SLR framework uses a model-free characterization of phase variations such that each state of phase variation needs to be specified by an individual image.

In addition to physiologically induced phase variations, bulk motion can induce *B*_0_ field fluctuations and image phase variations as well. The SLR formulation automatically accounts for any spatially smooth phase variations between shot groups, so any motion induced phase variations are also compensated for to some degree. However, the phase variations at the intra-shot group level are not considered currently. Some recent work ^57^ has considered motion induced *B*_0_ variations to improve the performance of mcSENSE for anatomical imaging at 7T. This method uses a physics-inspired *B*_0_ model which describes the *B*_0_ variation map as a linear combination of the pitch and roll rotation angles, and thus only two additional linear coefficient maps instead of an individual *B*_0_ variation map for each motion state need to be estimated. Similar approaches might be integrated to the current mcSLR framework by modeling motion induced intra-shot group phase variations with simple parametric models, which can further improve the motion robustness of mcSLR.

Inter-shot motion is modeled using a hierarchical strategy, which is decomposed into inter-shot group motion and intra-shot group motion. This motion decomposition makes it easier to estimate motion at different levels using different methods. Since the images for each shot group are available, the inter-shot group motion can be estimated straightforwardly by registration of images reconstructed from each shot group. While we chose the brain image registration tool FLIRT ^60^ in this work, any image registration algorithms/software packages can be used. Within each shot group, since it is challenging to perform image reconstruction for each individual subdivision, the intra-shot group motion is estimated by solving the joint image-motion optimization that exploits sensitivity encoding redundancy as in mcSENSE.

The proposed mcSLR reconstruction was also used in conjunction with the seg-CAIPI sampling pattern as in the previous SLR reconstruction ^43^, which has shown that the incoherent seg-CAIPI sampling can have a lower tSNR than coherent sampling when conventional SENSE reconstruction is used, due to the greater signal variations between adjacent k-space samples in seg-CAIPI acquired data. However, seg-CAIPI with SLR reconstruction can achieve a higher tSNR than the coherent sampling with SENSE reconstruction. Similarly, recent motion correction work ^50,51^ have demonstrated that incoherent sampling order has a higher sensitivity to motion and thus more image degradation with conventional SENSE reconstruction, but it can benefit the joint motion estimation and image reconstruction with mcSENSE. In this work, SENSE and SLR reconstructions show different types of motion artefacts in accordance with the seg-CAIPI trajectory. The interleaved ordering between different shot groups of the seg-CAIPI sampling results in incoherent mapping of motion cross the k-space for SENSE which reconstructs a single image from all shots, and thus leads to severe aliasing artefacts. In contrast, each interleave corresponding to a shot group samples k-space sequentially so that motion is still mapped coherently for SLR which reconstructs an image for each shot group, resulting in blurring artefacts.

Our previous SLR work^43^ has examined the trade-off between the number of shot groups and the amount of data available for each shot group in the motion-free regime, where using 4 shot groups (12 shots per shot group) was validated to be a good choice to boost tSNR when 1.8mm isotropic resolution and *R* = 2 × 2 was used. Considering the higher effective under-sampling factor even with perfect motion correction, fewer shots per shot group (<12) are not recommended, especially at higher spatial resolution or higher acceleration factors, even with ideal motion correction. However, this choice might may vary considering the accuracy of motion estimation. As discussed before, the trajectory of each shot group is still coherent, which does not benefit intra-shot group motion estimation. Thus, it is possible to improve the motion estimation accuracy for each motion state by performing only inter-shot group motion estimation, at the cost of lower motion parameter temporal resolution (e.g., 8 shots per shot group/motion state). In general, mcSLR provides a flexible framework that allows for motion compensation at different time scales, and explorations of different reconstruction choices (number of shots in each temporal subdivision and shot group) are encouraged in practice.

One limitation of the proposed method is that the 3D reconstruction problem is computationally demanding. The conventional 3D SLR reconstruction for each volume takes about 19 minutes per iteration on our computing cluster (6 core CPU with 96GB RAM), and the mcSLR reconstruction takes about 25 minutes per iteration, which corresponds to about 32% longer reconstruction time when the same prescribed number of iterations is used (30 iterations in this work). In addition, the initialization of motion parameters by mcSENSE also takes approximately 5.5 hours on average. In the current work, the numbers of iterations were determined empirically. Though the alternating minimization approach could potentially benefit from using more iterations for each subproblem, a relatively small number of iterations were used for ADMM and LM algorithms to solve subproblems 1 and 3 respectively for computational efficiency. Thus, the optimization of computational efficiency is a major task of future work. Specifically, the generic iterative reweighted annihilation filter (GIRAF) algorithm ^66^ which is SVD-free and matrix lifting-free is worthy of investigation ^67^. In addition, some automatic motion evaluation and classification procedures in the upstream pipeline could also be helpful to reduce the overall computational time of the time course by determining on which volumes mcSLR reconstruction is needed.

The current mcSLR reconstruction sometimes can suffer from minor under-sampling artifacts that have a slight ringing or checkerboarding effect, which indicates its limited capability to deal with worse conditioning of the reconstruction due to larger motion-induced larger gaps between k-space samples and imperfect coil sensitivities at the boundaries. Dealing with this may require better tuning of the joint estimation approach, or additional image reconstruction constraints (e.g., spatial total variation or wavelet), and will be a topic of future investigation.

## 5 Conclusion

In this work, a motion compensated structured low-rank (mcSLR) reconstruction method for robust 3D multi-shot EPI is proposed, which jointly models inter-shot motion and inter-shot phase variations. The proposed method improves the motion robustness of SLR framework which accounts for phase variations, and thus reduces motion artefacts and improves the temporal stability of 3D multi-shot EPI.

## Supplementary Material

Fig.S1-S7

Appendix
