# Supplementary material for: Motion Compensated Structured Low-rank Reconstruction for 3D Multi-shot EPI
Source: Magn Reson Med. Author manuscript; Available in PMC 2025 Oct 7. (PMC7618212; doi:10.1002/mrm.30019)
Supplement: Fig.S1-S7 [file EMS209277-supplement-Fig_S1_S7.docx]

**Supporting Information**


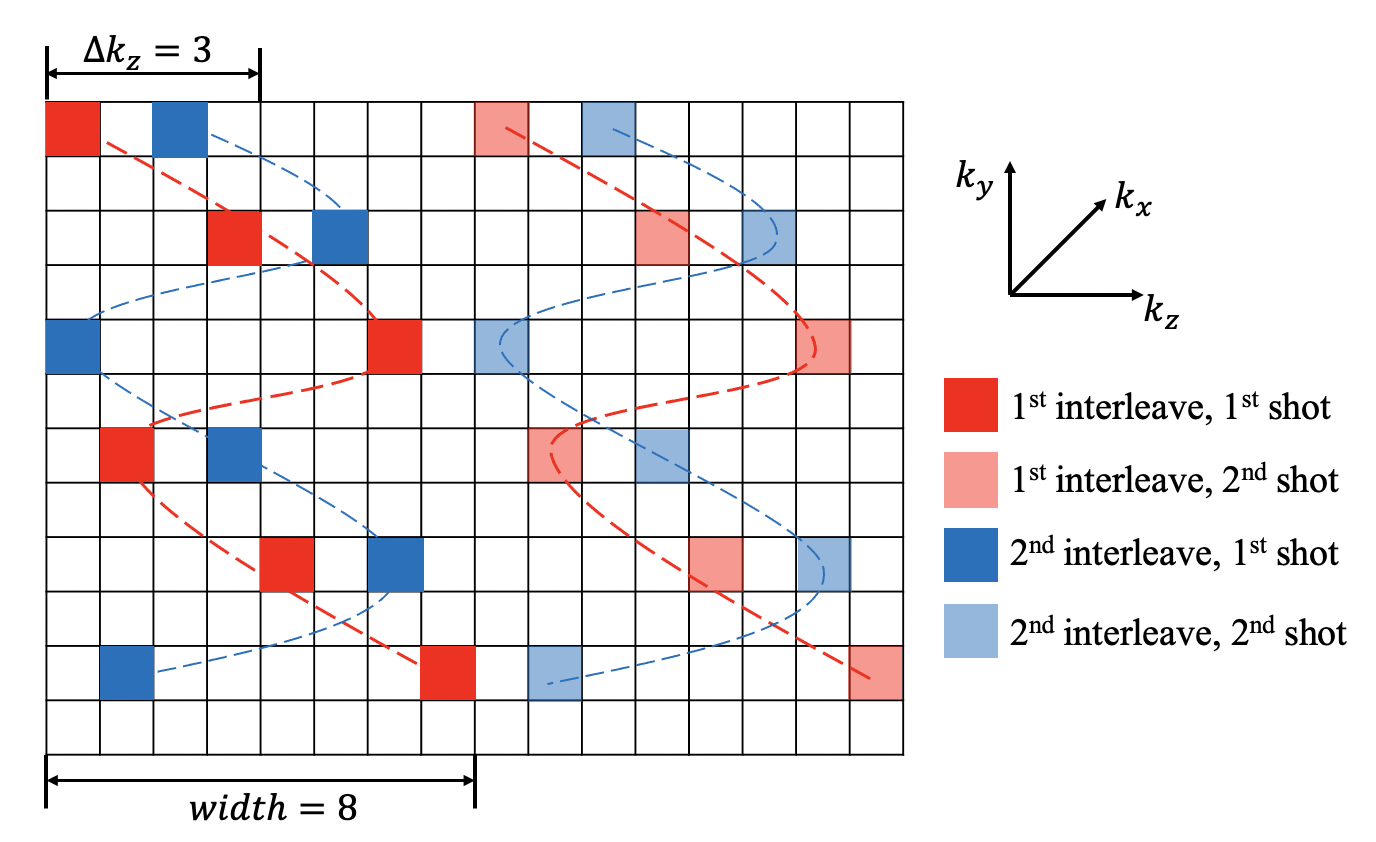


*Fig. S1. The seg-CAIPI sampling trajectory. The sampling trajectory is shown in*$k_{y}-k_{z}$ *plane and each solid square represents a readout line. All the readouts connected by a dashed line correspond to a single shot. Each shot is band limited along* $k_{z}$ *(*$width=8$*) with a blip size* $\Delta k_{z}=3$*. Interleaved ordering is used along* $k_{z}$*/shot dimension.* $R_{y}\times R_{z}=2\times2$*.*


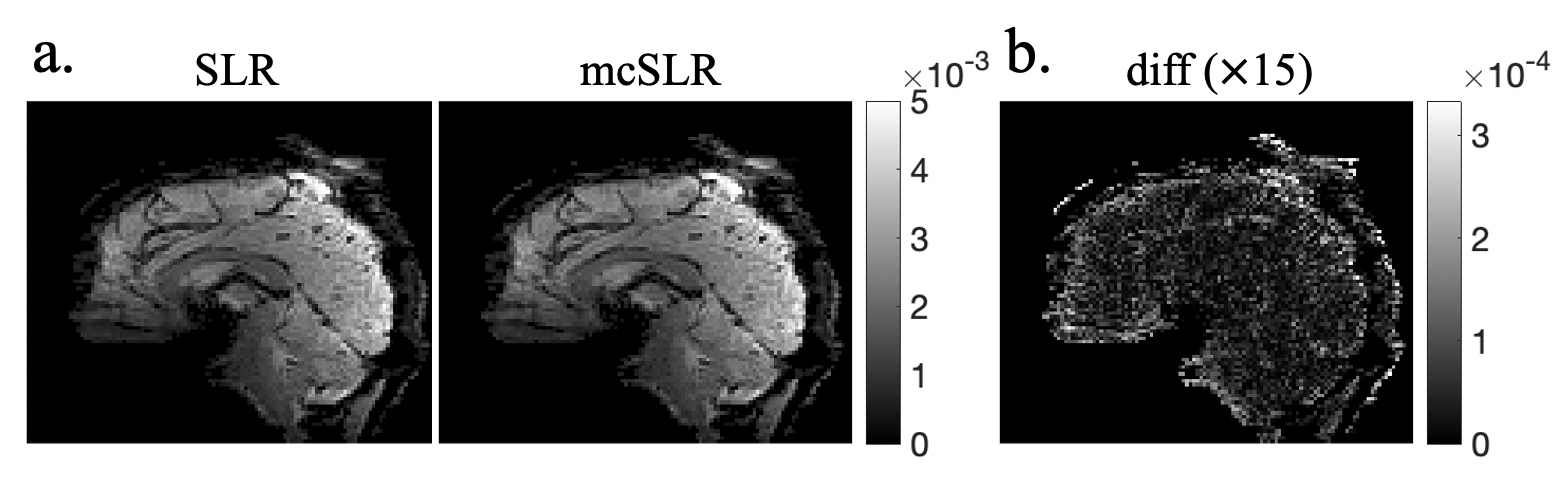


*Fig. S2. The comparison of SLR and mcSLR reconstructions on the motion-free dataset. a) The reconstructed images. b) The difference map between the two images in a). These two methods show comparable image quality and no significant differences.*


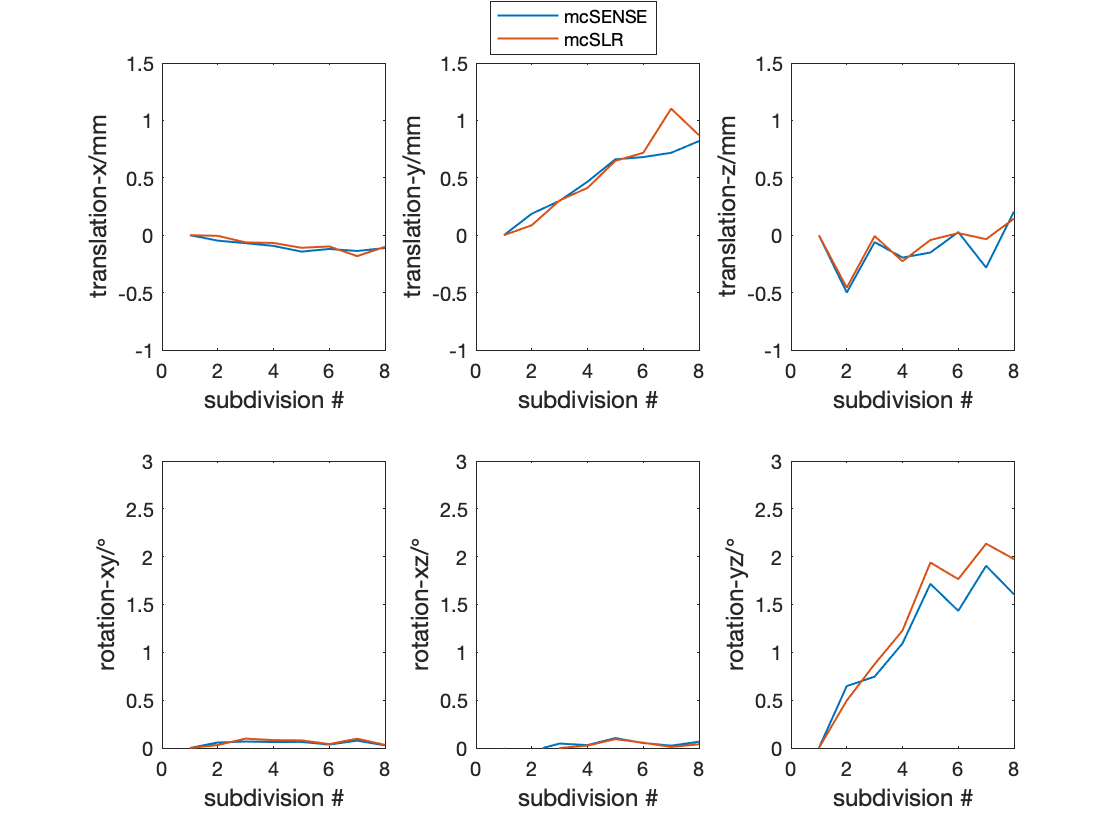


*Fig. S3. The motion estimates of mcSENSE and mcSLR reconstructions for the results shown in Fig. 4.*

*
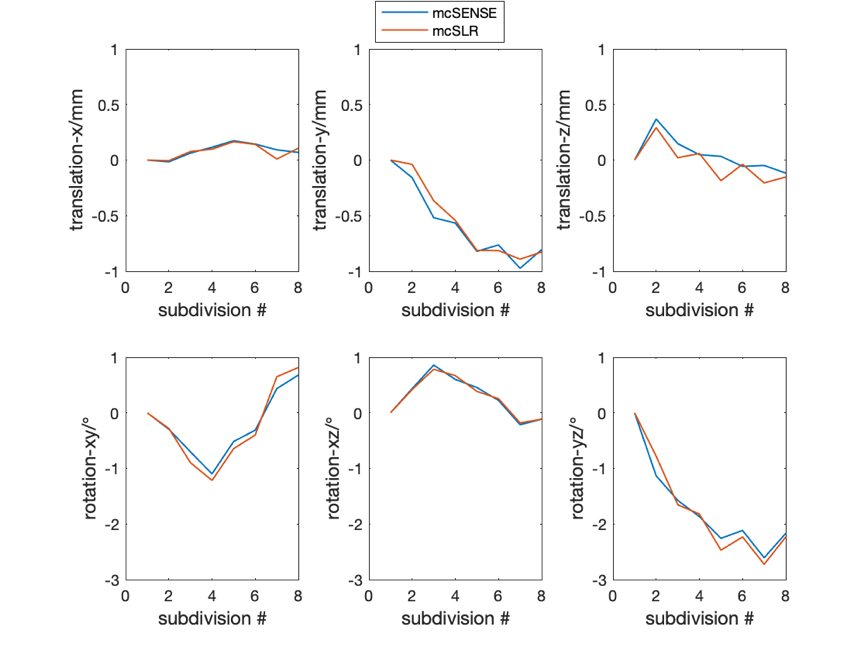
*

*Fig. S4. The motion estimates of mcSENSE and mcSLR reconstructions for the results shown in Fig. 5.*


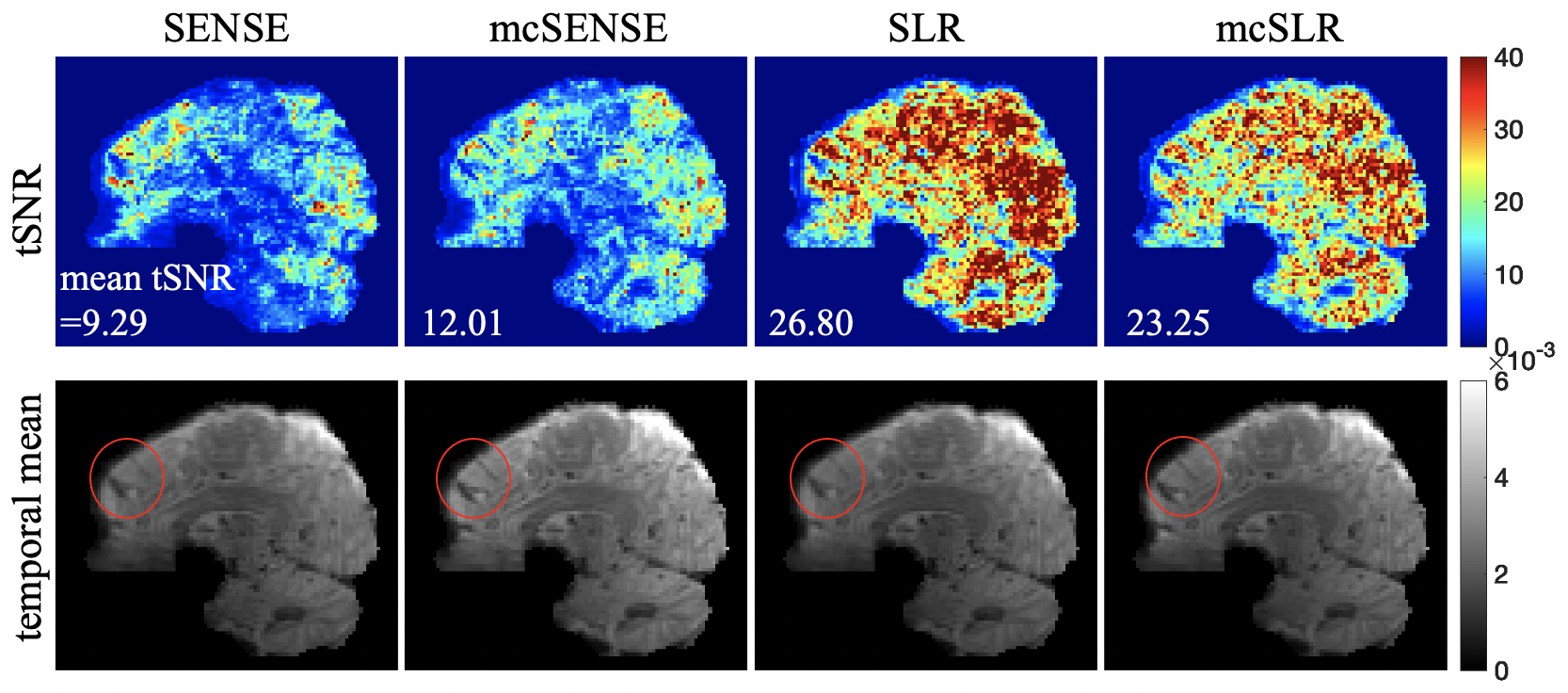


*Fig. S5. The tSNR comparison of different reconstruction methods. The tSNR maps and temporal mean magnitude images calculated across 15 volumes are shown. The mean tSNR value is shown in each tSNR map respectively. The red ellipses highlight effective blurring removal of mcSLR. The time course was registered with MCFLIRT prior to tSNR calculation. The mean tSNR of mcSLR is much higher than mcSENSE but slightly lower than SLR. However, the temporal mean image of SLR is blurrier than mcSLR (e.g., regions indicated by the red ellipse), which might lead to an inflated tSNR due to implicit spatial filtering effects.*


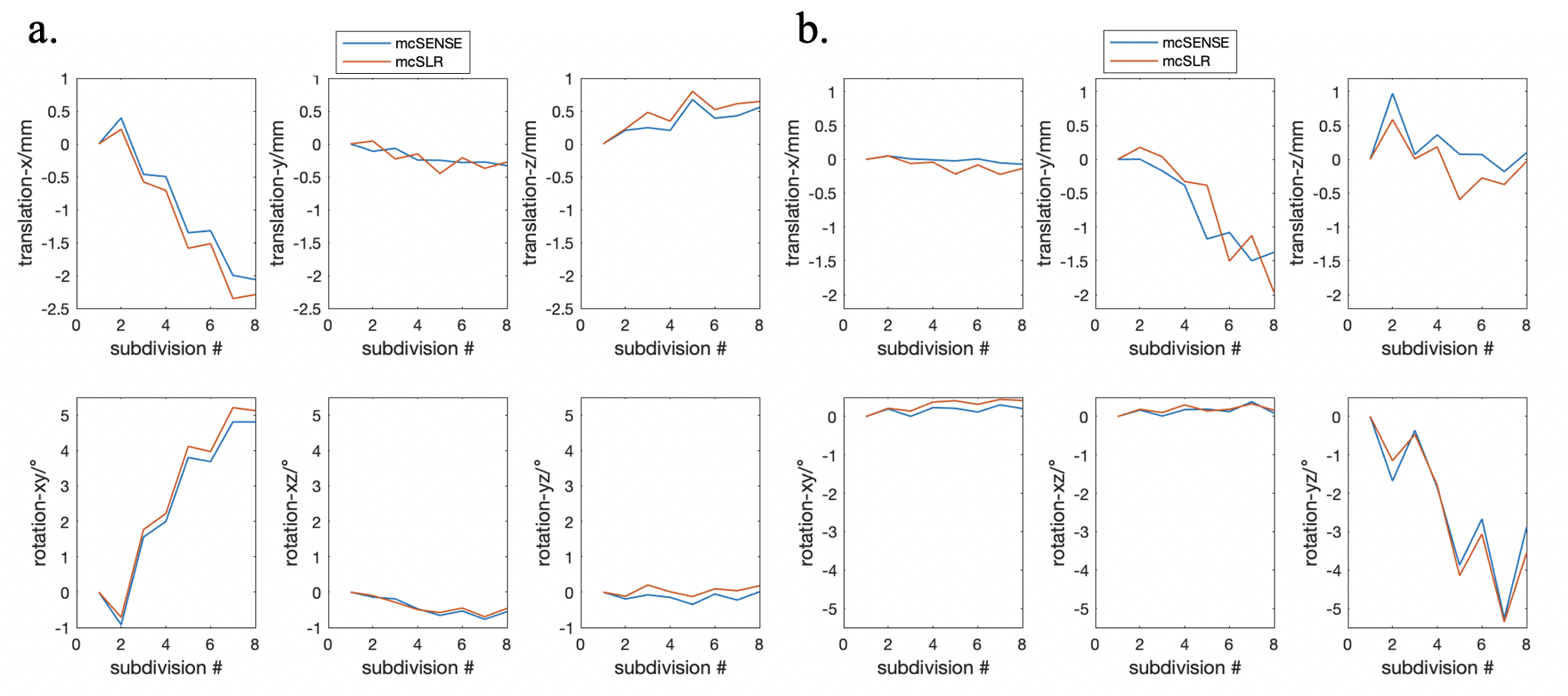


*Fig. S6. The motion estimates of mcSENSE and mcSLR reconstructions for the results shown in Fig. 9.*


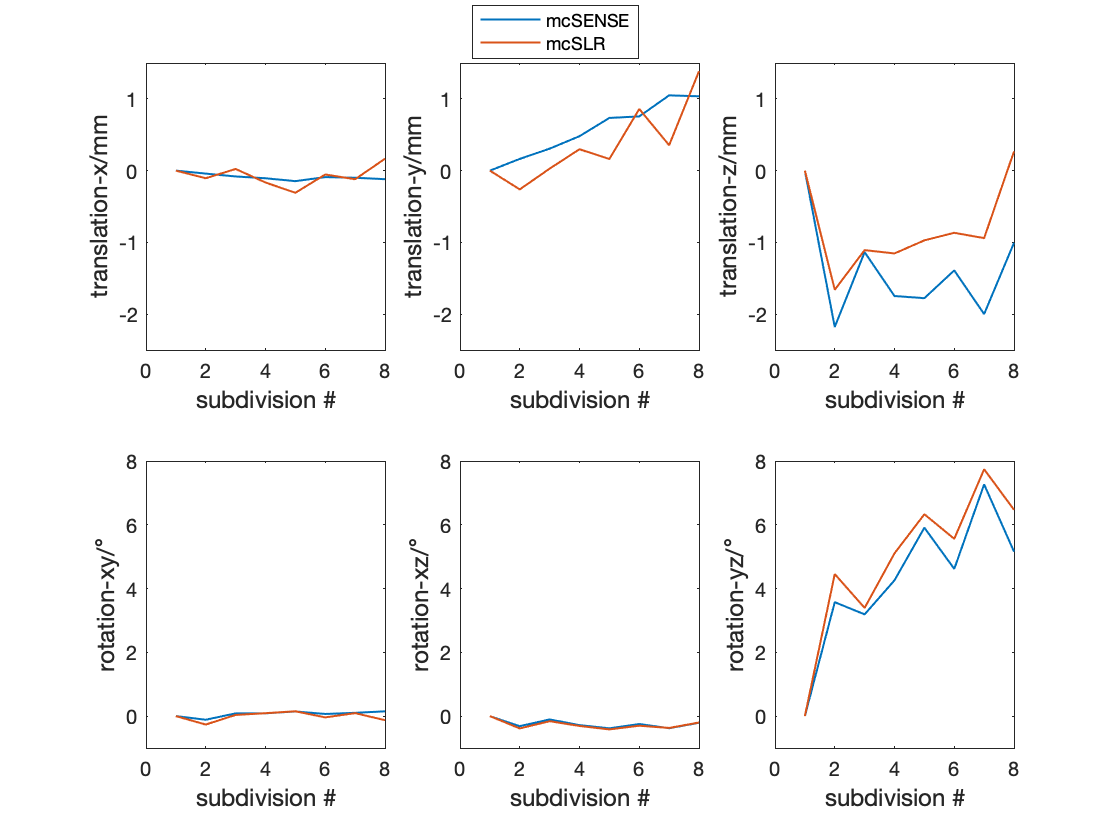


*Fig. S7. The motion estimates of mcSENSE and mcSLR reconstructions for the results shown in Fig. 10.*
