# Supplementary material for: Motion Compensated Structured Low-rank Reconstruction for 3D Multi-shot EPI
Source: Magn Reson Med. Author manuscript; Available in PMC 2025 Oct 7. (PMC7618212; doi:10.1002/mrm.30019)
Supplement: Appendix [file EMS209277-supplement-Appendix.docx]

**Appendix A**: Formulation of Motion Transformation

For the implementation of the rigid motion transformations $T_{intra}$ and $T_{inter},$ an interpolation-free representation is used as in mcSENSE^50,58^. This formulation decomposes a rotation into three consecutive shears to perform high quality rotations without the need of regridding. Thus, a 3D rigid motion transform with translation parameters [$\delta_{1},\delta_{2}, \delta_{3}$] and rotation parameters [$\theta_{1}, \theta_{3},\theta_{3}$] can be represented as a series of linear phase modulations in the hybrid $r-k$ space as:

|  | $T=F^{H}\Phi_{tra}F$ $\times F_{2}^{H}\Phi_{rot,\theta_{1}}^{tan}F_{2}F_{3}^{H}\Phi_{rot,\theta_{1}}^{sin}F_{3}F_{2}^{H}\Phi_{rot,\theta_{1}}^{tan}F_{2}$ $\times F_{3}^{H}\Phi_{rot,\theta_{2}}^{tan}F_{3}F_{1}^{H}\Phi_{rot,\theta_{2}}^{sin}F_{1}F_{3}^{H}\Phi_{rot,\theta_{2}}^{tan}F_{3}$ $\times F_{1}^{H}\Phi_{rot,\theta_{3}}^{tan}F_{1}F_{2}^{H}\Phi_{rot,\theta_{3}}^{sin}F_{2}F_{1}^{H}\Phi_{rot,\theta_{3}}^{tan}F_{1}$ | Eq. 4 |
| --- | --- | --- |

Where $F_{i}$ represents Fourier transform along the $i_{th}$ dimension. $\Phi_{tra}$ denotes the phase modulation in k-space corresponding to translation. $\Phi_{rot,*}^{tan}$ and $\Phi_{rot,*}^{sin}$ denote the phase modulations in the hybrid $r-k$ space that are used to implement the decomposed rotation. The entries of $\Phi_{tra}$, $\Phi_{rot,*}^{tan}$ and $\Phi_{rot,*}^{sin}$ are given as:

|  | $\phi_{tra}\left( k_{1},k_{2},k_{3} \right)=e^{-1j\left( \delta_{1}k_{1}+\delta_{2}k_{2}+\delta_{3}k_{3} \right)}$  $\phi_{rot,\theta_{1}}^{tan}(k_{2},r_{3})=e^{1j(tan(\theta_{1}/2)k_{2}r_{3})}\phi_{rot,\theta_{1}}^{sin}(k_{3},r_{2})=e^{1j(sin(\theta_{1}/2)k_{3}r_{2})}$  $\phi_{rot,\theta_{2}}^{tan}(k_{3},r_{1})=e^{1j(tan(\theta_{2}/2)k_{3}r_{1})}\phi_{rot,\theta_{2}}^{sin}(k_{1},r_{3})=e^{1j(sin(\theta_{2}/2)k_{1}r_{3})}$  $\phi_{rot,\theta_{3}}^{tan}(k_{1},r_{2})=e^{1j(tan(\theta_{3}/2)k_{1}r_{2})}\phi_{rot,\theta_{3}}^{sin}(k_{2},r_{1})=e^{1j(sin(\theta_{3}/2)k_{2}r_{1})}$ | Eq. 5 |
| --- | --- | --- |

Where $r_{*}$ is the coordinate in image space and $k_{*}$ is the coordinate in k-space.

Appendix B: The modified LM algorithm

The LM algorithm is an iterative method which can be viewed as a combination of gradient descent and Gauss-Newton methods. In each iteration, an increment of the motion parameters $\Delta T$ is calculated as:

|  | $\Delta T=-\left( wI_{d}+J^{H}J \right)^{-1}G$ | Eq. 6 |
| --- | --- | --- |

Where$w$ is the damping parameter which balances between gradient descent and Gauss-Newton methods. $I_{d}$ is the unit matrix. $J$ is the Jacobian matrix and$J^{H}J$is an approximation ofthe Hessian matrix. $G$ is the gradient of the cost function. When $w$ is very big, $\Delta T$ is determined by $G$ as in gradient descent method. When $w$ is small, $\Delta T$ is determined by ${(J^{H}J)}^{-1}G$ as in Gauss-Newton method. The cost function of potential new estimate $\hat{T}+\Delta T$ is compared to the cost function of the current estimate $\hat{T}$, and $\hat{T}+\Delta T$ will be accepted as the new estimate only if it has a lower cost function value. $w$ is updated automatically as follows: when the cost function value of $\hat{T}+\Delta T$ gets smaller, $w$ will be reduced in next iteration, otherwise it will be increased. A modification on the LM algorithm is also presented in this work such that instead of a scalar damping parameter, a vector $w$ in accordance with the dimensionality of the motion parameter (6 for rigid motion) is used. It is observed that this modification leads to a significant improvement in simulations, and a validation of this modification is shown in the Experiments section.
